# Mapping the overdose crisis: 6 locations using open medical examiner data

**DOI:** 10.1093/jamiaopen/ooaf140

**Published:** 2025-10-30

**Authors:** Daniel R Harris, Nicholas Anthony, Kelly A Keyes, Chris Delcher

**Affiliations:** Institute for Pharmaceutical Outcomes & Policy, Department of Pharmacy Practice and Science, College of Pharmacy, University of Kentucky, Lexington, KY 40508, United States; Institute for Biomedical Informatics, University of Kentucky, Lexington, KY 40508, United States; Institute for Pharmaceutical Outcomes & Policy, Department of Pharmacy Practice and Science, College of Pharmacy, University of Kentucky, Lexington, KY 40508, United States; Institute for Biomedical Informatics, University of Kentucky, Lexington, KY 40508, United States; Investigations Program, RTI International, Center for Forensic Science Advancement and Application, Research Triangle Park, NC 27709, United States; Institute for Pharmaceutical Outcomes & Policy, Department of Pharmacy Practice and Science, College of Pharmacy, University of Kentucky, Lexington, KY 40508, United States

**Keywords:** geographic mapping, datasets as topic, coroners and medical examiners, drug overdose

## Abstract

**Objective:**

Medical examiners and coroners (ME/C) oversee medicolegal death investigations which determine causes of death and other contextual factors that may have influenced a death. We utilize open data releases from ME/C offices covering 6 different geographic areas to demonstrate the strengths and limitations of ME/C data for forensic epidemiology research.

**Materials and Methods:**

We use our novel geoPIPE tool to establish a pipeline that (a) automates ingesting open data releases, (b) geocodes records where possible to yield a spatial component, (c) enhances data with variables useful for overdose research, such as flagging substances contributing to each death, and (d) publishes the enriched data to our open repository. We use results from this pipeline to highlight similarities and differences of overdose data across different sources.

**Results:**

Text processing to extract drugs contributing to each death yielded compatible data across all locations. Conversely, geospatial analyses are sometimes incompatible due to differences in available geographic resolution, which range from fine-grain latitude and longitude coordinates to larger regions identified by zip codes. Our pipeline pushes weekly results to an open repository.

**Discussion:**

Open ME/C data are highly useful for research on substance use disorders; our visualizations demonstrate the ability to contextualize overdose data within and across specific geographic regions. Furthermore, the spatial component of our results enables clustering of overdose events and accessibility studies for resources related to preventing overdose deaths.

**Conclusions:**

Given the utility to public health researchers, we advocate that other ME/C offices explore releasing open data and for policy makers to support and fund transparency efforts.

## Background and significance

The substance use and overdose crisis in the United States is continuously evolving and has entered a “third wave” with rising trends of polysubstance use, encompassing both illicitly manufactured opioids and stimulants.^1^ Downward trends in fentanyl overdoses are encouraging yet signals of an additional wave of fatal overdoses involving methamphetamine and cocaine use are growing.^2^ The economic burden of opioid use disorder and opioid-related overdoses is estimated to be over 1 trillion dollars, and localized data are needed to inform public policy and investment into prevention and targeted response activities.^3^ Publicly available dashboards on overdose are one such investment where community leaders, public health agencies, and citizens can access state and local trends. These dashboards can be more targeted to response efforts specifically designed for the overdose crisis, such as those developed by the HEALing Communities Study.^4–7^ Some states, counties, and communities go one step beyond making a dashboard publicly available and also make overdose death investigation data itself openly available; localized open data efforts are highly useful for substance use disorders research because they offer individual levels of detail not available otherwise and flexibility to analyze information beyond dashboard constraints.^8^

The United States Centers for Disease Control and Prevention (CDC) has published standardized fatal overdose data as part of CDC WONDER (Wide-Ranging Online Data for Epidemiologic Research), which pioneered released data specifically for epidemiologic research^9^; despite its inherit usefulness in studying overdoses, the utility of CDC WONDER in localized practice may be limited by data suppression rules triggered by small counts generated from county-level queries.^10^^,^^11^ CDC also operates the State Unintentional Drug Overdose Reporting System (SUDORS) which ingests, but does not disseminate, individual-level data from participating states and ME/C offices to support surveillance of overdose through high-level dashboards and reports.^12^ These datasets are useful for exploring and reporting regional trends, demographics, and population circumstances of overdose occurrences but are not available as public use files beyond aggregated reports. Individual level data from medical examiner and/or coroner (ME/C) offices describe in greater detail the nuances of a death investigation, including autopsy results, toxicology, medical history, and other contextual factors of the death scene, including specific locations such as the bodily injury site.^13^ Although ME/C reports may provide more detail than the processed data disseminated by federal systems, their utility in research is challenged by a lack of data standardization, poor accessibility due to privacy concerns, and reliance upon unstructured text fields.^14^^,^^15^ In 2022, the CDC established the Collaborating Office for Medical Examiners and Coroners (COMEC), which provides support to ME/C and promotes collection, automation, and distribution of data produced by ME/C investigations.^16^

The principles of open data require data to be free to access, use, modify, and share where only stipulations intended to preserve data provenance and openness are permissible.^17^ More specifically, open data must have an open license or be in the public domain, accessible through the internet without charge, and be available in an open format that does not require proprietary tools to use. We previously demonstrated that open street address data are critical for geospatial analyses and privacy studies.^18^^,^^19^ The United States government adopted an open data portal in 2009 to better support transparency and general use of datasets created by federal, state, and local governmental agencies, including those in public health.^20^^,^^21^ Consequently, many states, counties, and cities have adopted open data portals.^22^^,^^23^ In this work, we describe data from 6 ME/C offices who pioneered the release of open data for the field of “applied forensic epidemiology.” We process each dataset using geoPIPE, our custom tool that extends existing open data by geocoding records and adding variables relevant to researchers interested in substance use research.^8^ We empirically demonstrate that open ME/C data allows researchers to construct a more comprehensive view of fatal overdoses at both individual and community levels, which is needed to develop and monitor public responses and interventions designed to mitigate the crisis.

## Materials and methods

We obtained open ME/C data from the Cook County Medical Examiner’s Office (Illinois), the Milwaukee County Medical Examiner’s Office (Wisconsin), the County of San Diego Department of the Medical Examiner (California), the Santa Clara County Medical Examiner-Coroner (California), the Connecticut’s Office of the Chief Medical Examiner, and the Sacramento County Coroner’s Office (California).^24–27^ We summarize facets about each dataset in Table 1. All datasets include reported age, race, and sex demographics; ethnicity is not available in Milwaukee County or Santa Clara County. Each open dataset varies by period covered, by the smallest geographic unit available (latitude/longitude coordinates, addresses, city, zip), and frequency of updates (daily, monthly, or annually). These updates, depending on jurisdictional policy regarding the release of pending cases, may impact already published records by replacing incomplete data with data based on finalized investigation notes or toxicology results. These offices are included in this study because they released all records as open data in a downloadable format that is freely available to the public; other counties such as Miami-Dade County in Florida have online portals that allows searching ME/C records on a case-by-case basis instead of having all records downloadable online.^28^ Three of the open datasets are released on the Socrata platform, a cloud-based open data platform,^29^ and 2 use ArcGIS Server, Esri’s enterprise geospatial information system platform that enables organizations to share maps, data, and geoprocessing tools across the web.^30^ Both of these platforms have an application programming interface (API) for connecting and querying data.^29^^,^^30^ Santa Clara’s platform is custom but allows for downloading data.

**Table 1.** Facets of 6 open datasets from ME/C offices.

	Cook County, Illinois	Milwaukee County, Wisconsin	San Diego County, California	Connecticut	Santa Clara County, California	Sacramento County, California
**Time period**	2014-now	2002-now	1997 -now	2012-2024	2018-now	2019-now
**Frequency**	Daily	Daily	Monthly	Annually	Daily	Daily
**Smallest geographic unit**	Lat/Lng	Address	Zip/City	Lat/Lng	Lat/Lng	Zip
**Addresses available**	No	Yes	No	Yes	No	No
**Platform**	Socrata	ESRI ArcGIS	Socrata	Socrata	Custom	ESRI ArcGIS
**Demographics available**						
** Age**	Yes	Yes	Yes	Yes	Yes	Yes
** Sex**	Yes	Yes	Yes	Yes	Yes	Yes
** Race**	Yes	Yes	Yes	Yes	Yes	Yes
** Ethnicity**	Yes	No	Yes	Yes	No	No

We processed each office’s data using our open data pipeline tool, geoPIPE.^8^ We previously used geoPIPE to support hotspot analysis of opioid-related deaths from the Cook County Medical Examiner’s Office, to analyze proximity of overdose deaths to pharmacies,^8^ to examine opioid overdoses during the COVID-19 stay-at-home order period, and to look at xylazine-involved fatal overdoses.^31^^,^^32^ geoPIPE is designed to derive key variables from open data, such as latitude and longitude from multiple address types and specific drugs mentioned in long lists of complex, multiple substances found in the cause of death fields. Latitude and longitude are calculated from geocoding the raw address data available for resident and injury locations where available; these coordinates also assist in spatially joining the data to land use features (eg, park designations), census demographics, and high-risk locations in the built environment (eg, motels). The linkage to other publicly available datasets, such as data from the US Census Bureau, supports epidemiological rates, accelerating translational research on substance use research and ecological studies on area-level social determinants of health.

Specific substances are extracted from the cause of death field using our drug identification tool that leverages a custom lexicon of drugs and matches items in the cause of death field using Jaro-Winkler string similarity to specific drug names and drug types.^33^ Our drug lexicon includes both therapeutic medicines implicated in fatal overdoses (eg, oxycodone) and illicit substances (eg, illicitly manufactured fentanyl). Our drug dictionary contains synonyms for collapsing different variations of phrases into the same type of match, such as collapsing fentanyl with its scientific name, N-phenyl-N-[1-(2-phenylethyl)-4-piperidinyl]-propanamide. The output of our extraction is converted into binary indicator flags signaling the presence of each drug as a variable that is appended to the original dataset; this simplifies enumerating specific drugs or combinations of drugs present at death. We also created indicator variables for drug classes, such as opioids and stimulants, to support aggregation of drug data; drug lists for opioid and stimulant classes are found in our GitHub code repository as the default definitions, which may be adjusted or expanded.^8^^,^^34^ We manually annotated 20 randomly selected cases from each location (120 total) to identify the presence of alcohol, stimulants, opioids, benzodiazepines, and anti-depressants. Two subject-matter experts acted as annotators and independently labeled each case as having these drug classes; we use Cohen’s Kappa score to measure agreement. Consensus was manually reached after reviewing disagreements to determine ground truth labels. We compare our pipeline’s extractions with our human annotations using sensitivity, precision, negative-predictive value, and F1-scores.

The pipeline feature to customize and expand the drug data is critical as the number of ingested drugs found at the time of death are increasing in number and in diversity (eg, fentanyl analogs).^35^ This drug class aggregation is used in Table 2. Our code repository includes weekly data releases containing processed versions of each dataset and a merged dataset containing drug matches and similarity scores for each location. Each dataset is standardized in “wide” format, where cause of death and address fields are converted to indicator variables.^34^ This creates the final 54 columns that remain consistent across all 6 data sources. We standardized column names wherever possible using commonalities listed in Table 1. For instance, while 2 counties lack ethnicity data, all sources include a standardized “race” variable. Some location-specific variables have limited utility since our text and spatial analyses provide superior alternatives—Cook County’s “district” variable, for example, is superseded by our geocoding process that generates US Census Bureau regions for sub-county analysis.

**Table 2.** Overdose data summarized.

	Cook County, Illinois	Milwaukee County, Wisconsin	San Diego County, California	Connecticut	Santa Clara County, California	Sacramento County, California
**Population (2022)**	5 109 292	918 661	3 276 208	3 626 205	1 870 945	1 585 055
**Scope**	All Deaths	All Deaths	All Deaths	Accidental Drug Deaths	All Deaths	Accidental Fentanyl Deaths
**Pre-existing structured fields on overdose**	Opioid Related	None	Opioid Related	All records are overdose	None	None
**No. of Cause of death fields (eg, columns)**	5	3	3	1	1	1
**Pending status available**	Yes	No	Yes	Yes	Yes	Yes
**Deaths**						
** All records**	78 168	65 612	79 378	10 654	24 901	1111
** Overdose-related text matches**	3078	1110	1880	1290	753	430
** Opioid-related text matches**	2477 (80.5%)	757 (68.1%)	1026 (54.6%)	1189 (92.2%)	315 (41.8%)	420 (38.7%)
** Stimulant-related text matches**	1788 (58.1%)	738 (66.5%)	1,115 (59.3%)	727 (56.4%)	452 (60.0%)	269 (24.2%)
**Drug mentions**	95 319	25 362	56 955	27 690	11 843	1983

The steps of our pipeline are abstracted and are controlled by a configuration file for each dataset; this configuration file contains the data’s URL for downloading, controls if geocoding or text processing occurs by specifying what variables in the original dataset are to be used for geocoding and drug extraction. Only Cook County, Milwaukee County, and Santa Clara County have address data, so geocoding is turned off for the other locations. We have automated the ingestion and processing of open data as part of our pipeline and published our extensions as derived open datasets that are refreshed weekly.^34^

## Results

We use the processed results of each dataset with geoPIPE to describe fatal overdoses in each location and to demonstrate the research applicability of open ME/C data. Although each dataset is similar, we summarize key characteristics relevant to overdose in Table 2; population is as reported as of the 2022 estimates from the US Census Bureau.^36^ The scope of Connecticut’s data is slightly different since it represents an entire state and is limited to their accidental drug deaths data. We processed “All Death” records available in the raw data to identify overdose-related encounters by matching records with our drug dictionary (Table 2). The scope of “All Deaths” is limited by jurisdiction and state law; only deaths under the jurisdiction of these specific offices are included in their open data. Cause of death strings were consistently formatted as short phrases across all data sources, though field structure varied. Cook County provided 5 separate fields for primary, secondary, and specific causes; Milwaukee County and San Diego each used 3 fields; and all other sources used a single field. Fields were specified for processing in the JSON configuration file for weekly analyses. Cook County, Illinois and San Diego County, California had preexisting structured fields specific to indicate specific types of overdoses to accompany the cause of death flags; we add our custom flags to all data sources to enable visualization and analyses per substance. Content of the cause of death fields were consistent across data sources as only 13 phrases were exclusively used in a single data source; for example, only Cook County used phrases “4-FBF” for 4-Fluorobutyrfentanyl and “4-FiBF” for 4-fluoroisobutyryl fentanyl. This consistency across data sources suggests performance of drug extraction for our indicator variables is comparable to our initial study.^8^

We extract evidence of both opioid class overdoses while also detecting specific opioids that may be explicitly listed as contributing to the death in the related cause of death text fields. We evaluated performance of our primary indicator variables for alcohol, stimulants, opioids, benzodiazepines, and anti-depressants; this evaluation did not consider pre-existing fields on overdose (Table 2) and was strictly limited to drugs identified using text fields for cause of death, which is what our pipeline uses for automation. Cohen’s kappa was 0.98 between our 2 human annotators, which indicated almost perfect agreement. There was disagreement about kratom, an herbal product with stimulant-like effects at low doses and opioid-like effects at higher doses^37^; our ME/C data do not quantify amount detected in toxicology results.

We compared our tool’s performance per class by computing per-class for prevalence, sensitivity, precision, negative predictive value (NPV), and F1; we computed macro-averaged performance by averaging across classes (Table 3). The indicator variables for these cases and classes that were generated by our pipeline aligned with performance for ethanol, stimulants, opiates, and benzodiazepines. However, our tool did not detect the presence of 2 anti-depressants due to them being absent from our drug dictionary. For comprehensiveness, users of our data releases need to use both pre-existing structured fields for overdose and our text-based indicator variables.

**Table 3.** Performance evaluation of substance indicator variables on random sample (*N* = 120) of annotated cases.

Indicator	Kappa	Prevalence	Sensitivity	Precision	NPV	F1
**Ethanol/alcohol**	0.95	0.30	1.0	1.0	1.0	1.0
**Stimulant**	0.98	0.40	1.0	1.0	1.0	1.0
**Opiate**	1.0	0.75	1.0	1.0	1.0	1.0
**Benzodiazepines**	0.93	0.15	1.0	1.0	1.0	1.0
**Anti-depressants**	1.0	0.03	0.5	1.0	0.98	0.67
**Macro average**	0.98	0.33	0.90	1.0	0.99	0.93

**Figure 1.**
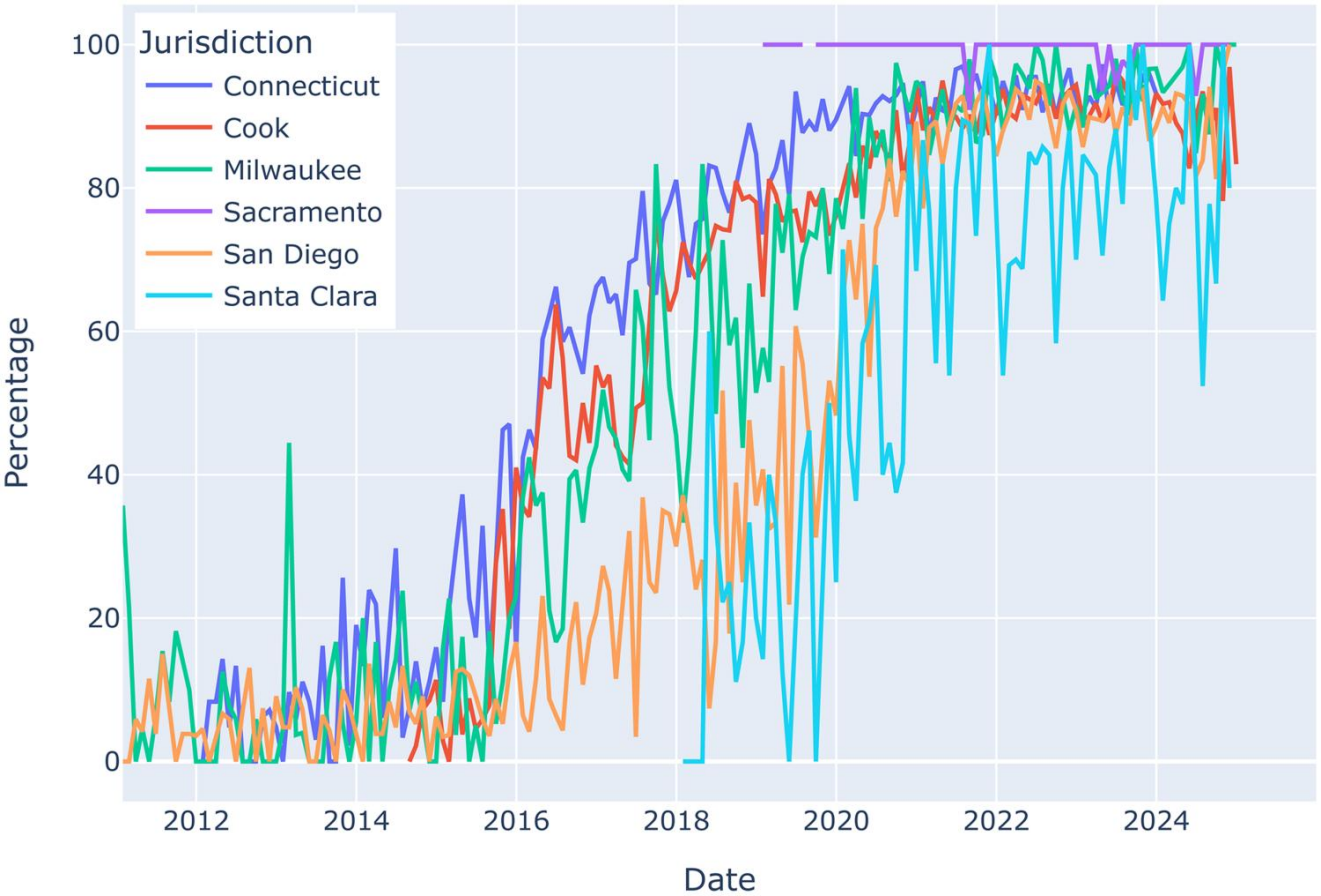
Percent of opioid overdoses containing fentanyl over time (monthly, January 2012-September 2024).

Our pipeline also yields indicator variables for individual drugs. Figure 1 shows the percentage of opioid overdoses containing fentanyl over time, which is increasing for all locations. Prior to 2016, it was rare for the 4 jurisdictions with data available to exceed 40% fentanyl-involved opioid overdoses for any given month. By 2022, opioid overdoses involving fentanyl consistently exceeded 80% which aligns with national trends.^38^ Note that Sacramento’s dataset only reports on fentanyl overdoses by design and 37.8% of these records contained specific opioid-related indicators, such as fentanyl, as part of their cause of death text fields (420 out 1,111); the remaining 62.2% are known to be fentanyl from structured data fields. Additionally, rapid declines observable towards the end of the time series data indicates incomplete or pending investigation records that likely will be updated once toxicology results are available. This is a strong limitation of open data from the ME/C community as data are often continuously updated or modified in retrospect pending the results of different aspects of the overdose investigation. Open data should be annotated so that users understand these delays and avoid misinterpreting such changes as true epidemiologic trends; for example, the Cook County ME/C data contains a field indicating whether or not the case is pending; Table 2 describes which locations have a pending status available.

**Figure 2.**
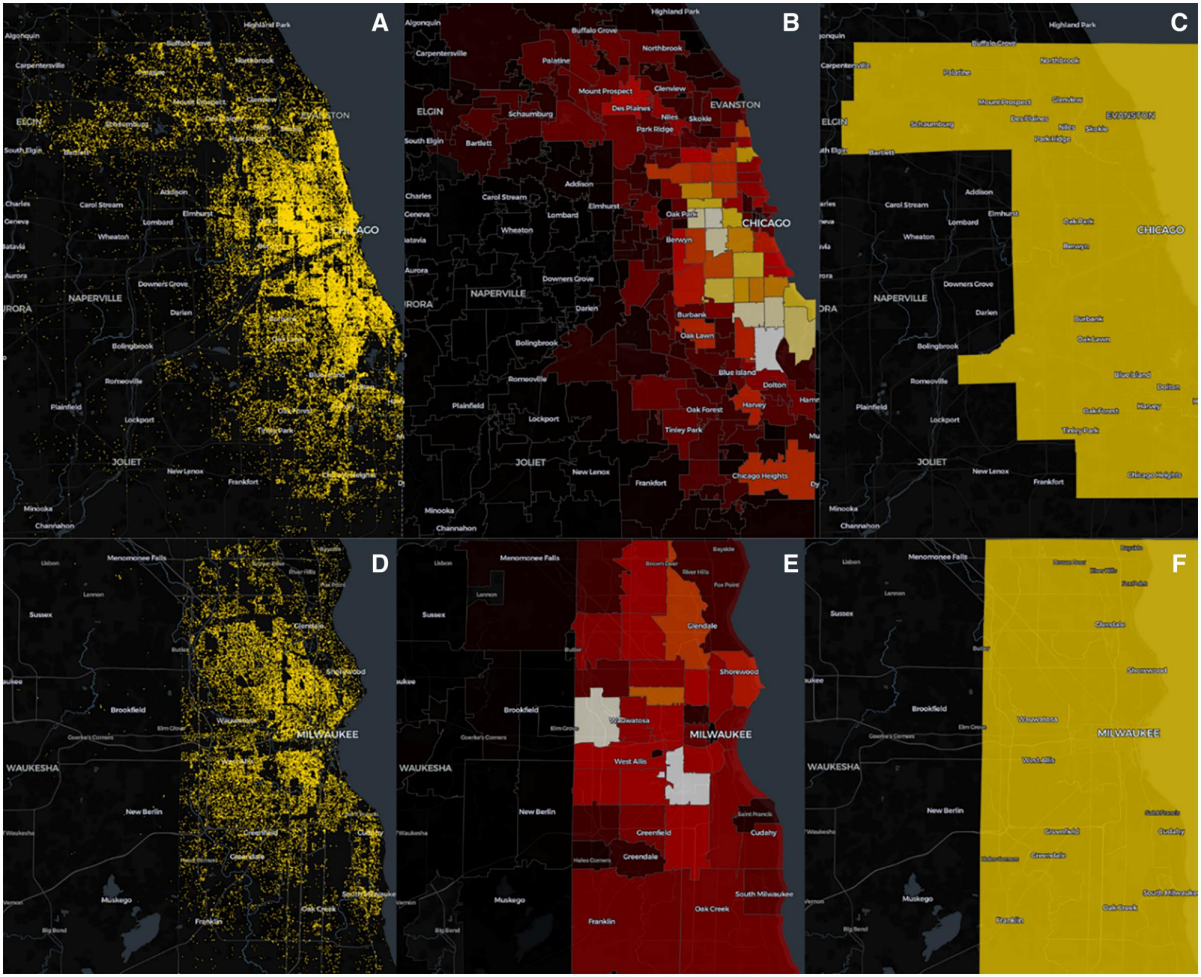
Opioid overdoses visualized using point coordinates, zip codes, and counties in Cook County, Illinois (A, B, C) and Milwaukee County, Wisconsin (D, E, F).

Due to differences in the geographic resolution available in each dataset, geospatial analyses may not be directly comparable. For example, Cook County and Santa Clara have death records recorded at the latitude and longitude level which allows aggregation into any administrative or arbitrary boundary regardless of size. Figure 2 shows opioid overdoses visualized for Cook County, Illinois (top row) and Milwaukee County, Wisconsin (bottom row); the geographic resolution clearly varies between individual records visualized by using latitude and longitude coordinates (A, D), zip codes (B, E), and or counties (C, F). Note that Sacramento County and San Diego County cannot be visualized as points since zip codes are their smallest geographic unit.

**Figure 3.**
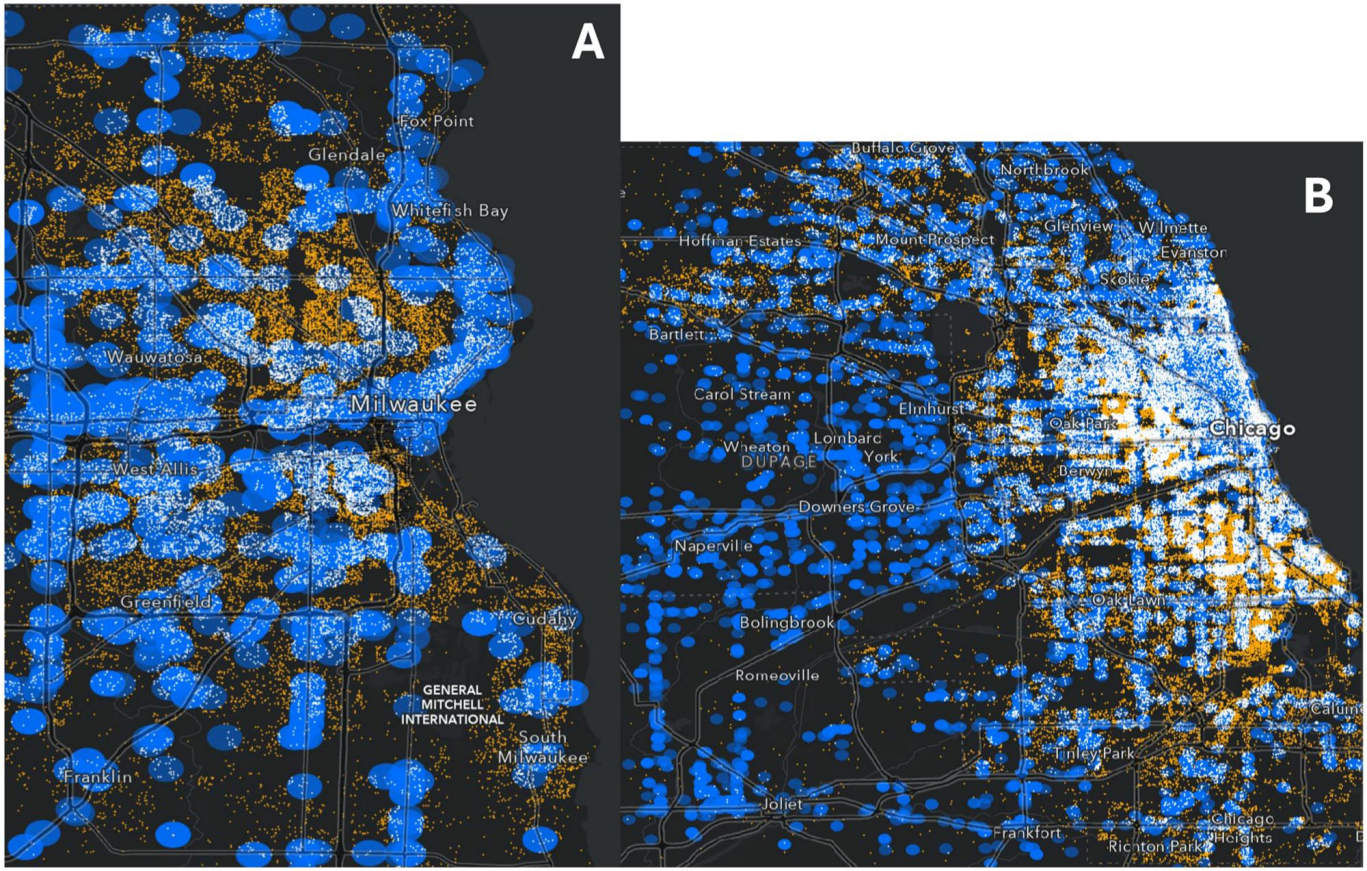
Overdose deaths and proximity to pharmacies in Cook County, Illinois (A) and Milwaukee County, Wisconsin (B): blue regions are 1/3-mile buffer zones around a pharmacy; white dots represent overdoses within the buffer zone for a pharmacy and orange dots are overdoses outside of any zone.

Figure 3 demonstrates the value of coordinate data by examining a case study of opioid overdoses and their proximity to pharmacies. Pharmacies are one source of naloxone, the highly effective opioid overdose reversal drug in which pharmacies may play a large distribution role.^39^ Pharmacy locations were sourced from the Drug Enforcement Administration’s Automation of Reports and Consolidated Orders System (ARCOS), which is available nationally.^40^^,^^41^ We created circular buffer zones with a 1/3-mile radius around all pharmacies in Cook County, Illinois and Milwaukee County, Wisconsin. We colored opioid overdoses based on whether the location of the overdose is within these buffer zones (white) or outside (orange). This visualization demonstrates the vast number of opioid overdoses occurring in regions with limited spatial proximity to pharmacies, which could be used for community planning and naloxone distribution strategies by public health officials.

## Discussion

Although each location publishes data in its own localized format, our geoPIPE tool was able to standardize input and process each raw dataset, add variables specific to deaths related to substance use disorders, and push the results back to our data repository. For each data source, we generate fine-grain details for each record representing individual decedents. Drug identification uses the cause of death fields available, with extensive drug names, and enables analysis not immediately possible in the original open dataset; this yields compatible data across locations and enables harmonious multi-site analyses examining contributing factors in overdoses. All locations had geographic data and demographic data reported for age, sex, and race, which are needed for research examining social determinants of health (SDOH).^42^ Furthermore, for SDOH, records were associated with a specific location, although precision varied from coordinate level to zip code or city level. The latter creates a significant limitation, as these types of datasets cannot support distance-based analyses or aggregations smaller than zip codes. To some degree, SDOH can be inferred from jurisdictions that provide the injury location type (eg, shelter, vacant house). An additional key difference across the datasets is the speed of updates which ranged from daily to annual, which may limit the utility of the data. For example, opioid-related dashboards require recent data to be effective for immediate community interventions.^7^

Each location offers a unique perspective of overdose deaths. The Connecticut data are the only state-wide dataset which improves its reach and utility; conversely, the scope is limited to accidental drug deaths only which were determined by a scene investigation, autopsy, and toxicology analysis. The Cook County, Illinois data contain the city of Chicago and represent the second most-populated county in the United States, thus providing a daily snapshot of overdose activity and trends that may signal early outbreaks, show impact on smaller sub-communities, or help underscore differences between urban and rural opioid supply factors.^43–45^ Sacramento County’s data were limited to fentanyl-involved deaths, which underscores the importance of fentanyl and its impact on overdoses increasing in Western states.^46^^,^^47^ Records in San Diego County spanned from 1997 to current, which was the longest period covered by any location. Santa Clara’s data were made available using a custom platform, instead of a commercial product; this allowed us to test the generalizability of geoPIPE and its ability to read different types of data sources. Records from Milwaukee County contained address locations as text fields, which required geocoding by geoPIPE; other locations such as Cook County also contained address locations but were available alongside spatial coordinates. Despite having spatial coordinates, we geocoded records in-house to confirm accuracy and fill in missing or incomplete records; we previously described the positive impact of this preprocessing on data quality.^8^

The importance of the open data released by these offices is highlighted by their influence on overdose research which has mostly either explored emerging trends or analyzed the spatial context.^31^^,^^32^^,^^48–52^ This work and the related work would either not be possible or significantly more difficult without open data. Our work enhances existing open data by adding important variables for substance use disorders research. An important limitation of our study is that not all deaths from our locations fall under the jurisdiction of the location’s ME/C office; only deaths requiring investigation from these specific offices are included in their open data, which typically includes deaths from overdose as best practice indicated by the National Association of Medical Examiners.^13^ All of our locations use medical examiner systems; there is variation state to state in specificity of overdose documentation^53^ and coroner systems are known to yield more unspecified overdoses,^54^ which may limit our tool’s utility for those areas. State laws in each of these office’s locations allow the ME/C to release public records on death investigations but aspiring ME/C offices from other jurisdictions wishing to release open data would need to navigate and verify applicable regional statutes. Geocoding yields exact latitude and longitude coordinates which are inherently sensitive data points; the locations discussed in this study all consider death records a matter of public records and leverage open data portals as a mechanism of supporting inquiry of public records. Strategies exist to incorporate geospatial data safely into research studies^42^ and to obfuscate the original coordinates without destroying research utility, such as protecting linkages to neighborhood-level SDOH.^55^

Text fields are pivotal to identifying overdoses in other settings, such as emergency departments.^56^ Our text processing results are inherently limited by the completeness of the death record which may get updated in the raw data based on the investigation or toxicology results. If a substance is not detected nor documented by the ME/C, our methods are not applicable; conversely with polysubstance overdose, presence of a medication does not necessarily establish its role in causing death, as it may be present at therapeutic rather than toxic levels. Our indicator variables are also limited by the completeness of our drug dictionary uses for drug identification, as seen by having incomplete results for anti-depressants; we wish to explore integrating multiple sources of knowledge and controlled vocabularies in the future for drug identification. Our indicator variables assume any dosage, which impacts herbal products such as kratom, which acts as a stimulant at low doses and is opioid-like at higher doses.^37^ We did not have a systematic way of discovering open datasets and relied heavily on manual identification of open data portals using search engines and professional networks. In the future, we wish to automate the creation of specific visualizations and dashboards for near-real monitoring of overdose activity recorded by ME/C offices. Other data sources, such as prescription drug monitoring programs (PDMPs) or emergency department discharge data, could help contextualize trends observable in our open data; previous studies found unclear associations between either PDMP implementation or controlled-substance prescribing and overdose rates, further justifying analysis on the topic using real-world data.^57^^,^^58^ Given the weekly updates to our repository, this open data resource could support time-sensitive studies requiring rapid, actionable information. For instance, open ME/C data could serve as provisional early indicators of overdose fatalities, offering a faster alternative to official statistics that typically require several months to process through traditional data sources.^59^^,^^60^ Additionally, our repository could be used to analyze region-specific reporting lags to estimate when toxicology results and death records are finalized in different jurisdictions.

## Conclusions

We have demonstrated that open ME/C data can be systematically processed and enriched for use in substance use disorders research and intervention efforts. Each location publishing open data has provided a disparate glimpse of death investigations for their area; we show that the natural differences amongst these datasets are relatively straightforward to navigate. Our work suggests the need to survey the ME/C community to understand barriers to open data release and to identify additional offices willing to submit to an open data repository; if data for SUDORS is already collected and transmitted to the CDC for surveillance purposes, it may be suitable for an open data system. We hope our work will encourage other ME/C offices to explore releasing open data to benefit researchers, public health practitioners, and ultimately society itself as these data points are leveraged to support new findings and discoveries.

## Data Availability

Both code and data for this project are available online at https://github.com/UK-IPOP/open-data-pipeline.
